# Membrane expression of thymidine kinase 1 and potential clinical relevance in lung, breast, and colorectal malignancies

**DOI:** 10.1186/s12935-018-0633-9

**Published:** 2018-09-10

**Authors:** Evita G. Weagel, Weston Burrup, Roman Kovtun, Edwin J. Velazquez, Abigail M. Felsted, Michelle H. Townsend, Zachary E. Ence, Erica Suh, Stephen R. Piccolo, K. Scott Weber, Richard A. Robison, Kim L. O’Neill

**Affiliations:** 1 Department of Microbiology and Molecular Biology, Brigham Young University, 3142 Life Sciences Building, Provo, UT 84602 USA; 2 Department of Biology, Brigham Young University, Provo, UT USA; 3 Department of Biomedical Informatics, University of Utah, Salt Lake City, UT USA

**Keywords:** TK1, Surface expression, Membrane TK1, Thymidine kinase 1, Lung cancer, Breast cancer, Colon cancer

## Abstract

**Background:**

Lung, breast, and colorectal malignancies are the leading cause of cancer-related deaths in the world causing over 2.8 million cancer-related deaths yearly. Despite efforts to improve prevention methods, early detection, and treatments, survival rates for advanced stage lung, breast, and colon cancer remain low, indicating a critical need to identify cancer-specific biomarkers for early detection and treatment. Thymidine kinase 1 (TK1) is a nucleotide salvage pathway enzyme involved in cellular proliferation and considered an important tumor proliferation biomarker in the serum. In this study, we further characterized TK1’s potential as a tumor biomarker and immunotherapeutic target and clinical relevance.

**Methods:**

We assessed TK1 surface localization by flow cytometry and confocal microscopy in lung (NCI-H460, A549), breast (MDA-MB-231, MCF7), and colorectal (HT-29, SW620) cancer cell lines. We also isolated cell surface proteins from HT-29 cells and performed a western blot confirming the presence of TK1 on cell membrane protein fractions. To evaluate TK1’s clinical relevance, we compared TK1 expression levels in normal and malignant tissue through flow cytometry and immunohistochemistry. We also analyzed RNA-Seq data from The Cancer Genome Atlas (TCGA) to assess differential expression of the TK1 gene in lung, breast, and colorectal cancer patients.

**Results:**

We found significant expression of TK1 on the surface of NCI-H460, A549, MDA-MB-231, MCF7, and HT-29 cell lines and a strong association between TK1’s localization with the membrane through confocal microscopy and Western blot. We found negligible TK1 surface expression in normal healthy tissue and significantly higher TK1 expression in malignant tissues. Patient data from TCGA revealed that the TK1 gene expression is upregulated in cancer patients compared to normal healthy patients.

**Conclusions:**

Our results show that TK1 localizes on the surface of lung, breast, and colorectal cell lines and is upregulated in malignant tissues and patients compared to healthy tissues and patients. We conclude that TK1 is a potential clinical biomarker for the treatment of lung, breast, and colorectal cancer.

**Electronic supplementary material:**

The online version of this article (10.1186/s12935-018-0633-9) contains supplementary material, which is available to authorized users.

## Background

Lung, breast, and colorectal malignancies are the leading causes of cancer-related deaths in the world. These three cancers account for over 4.86 million cases diagnosed and over 2.8 million cancer-related deaths worldwide every year [1]. Thus, lung, breast, and colorectal cancers are a major health concern as over 11.7 million people are currently diagnosed and living with these diseases worldwide and represent a substantial economic burden in countries of all incomes [1, 2].

Despite efforts to improve methods of prevention, early detection, and treatments, survival rates for advanced stage lung, breast, and colon cancer remain low at 4%, 26%, and 13%, respectively [3]. Therefore, there is an urgent need to identify cancer-specific biomarkers for early detection and treatment of the leading cause of cancer-related deaths such as lung, breast, and colorectal cancers [4, 5].

Thymidine kinase 1 (TK1) is a nucleotide salvage pathway enzyme involved in cellular proliferation and considered an important tumor proliferation biomarker [6–9]. In serum, TK1 has been shown to be elevated in early events of malignancy, and thus, TK1 can serve as an early detection biomarker [9–11]. Moreover, serum TK1 has been found to be elevated in several hematological and solid tumors including breast, lung, colorectal cancer, among others, and high serum TK1 levels usually correlate with cancer grade and stage, increased T-values, and increased tumor size [7–9, 12–14]. Serum TK1 can be also used as a prognostic tool to monitor responses to chemotherapy or surgery [14, 15].

To further characterize TK1’s potential as a tumor biomarker, we evaluate TK1 as a potential immunotherapeutic target. In this study, we evaluate the expression levels of membrane TK1 on lung, breast, and colorectal cell lines using flow cytometry. We also show evidence that TK1 is localized on the surface of lung, breast, and colorectal cell lines. In addition, we evaluate TK1 expression levels in normal and malignant tissue to determine TK1’s clinical relevance. These results suggest TK1 as a potential immunotherapeutic target.

## Materials and methods

### Cell lines and cell culture conditions

Lung cancer cell lines NCI-H460 (ATCC^®^ HTB-1770™) and A549 (ATCC^®^ CCL-185™), breast cancer cell lines MCF7 (ATCC^®^ HTB-22™) and MDA-MB-231 (ATCC^®^ HTB-26™), and colon carcinoma cell lines SW620 (ATCC^®^ CCL-227™) and HT-29 (ATCC^®^ HTB-38™) were purchased from ATCC (Rockville, MD). NCI-H460 and HT-29 cell lines were grown in RPMI 1640 medium (Corning Life Sciences, VWR International, Radnor, PA) supplemented with 2 mM l-glutamine and 10% fetal bovine serum. MDA-MB-231, MCF7, and SW620 cell lines were grown in DMEM medium (Gibco, Thermo Fisher, Waltham, MA) supplemented with 4 mM l-glutamine and 10% fetal bovine serum. A549 cells were grown in DMEM/F-12 medium (Gibco, Thermo Fisher, Waltham, MA) supplemented with 4 mM l-glutamine and 10% fetal bovine serum. l-glutamine and fetal bovine serum were purchased from Thermo Fisher (Waltham, MA). The media was renewed every 2–3 days. For subculturing, cells were detached using Accutase (Stem Cell Technology, Vancouver, Canada) and seeded in 1:3 or 1:6 ratios. All cells were cultured at 37 °C with 5% CO_2_. All cell lines were authenticated by short tandem repeat (STR) analysis at the University of Arizona Genetics Core Facility during our study.

### Antibodies

We used three custom mouse monoclonal antibodies developed in our lab against TK1 (CB1, A72, and A74) and a commercially available rabbit monoclonal antibody against TK1 (ab91651) (Abcam, Cambridge, United Kingdom). CB1 binds to the C-terminal domain of TK1, specifically to the active domain. A72 and A74 are against an immunodominant region not on the TK1 C-terminal domain. These antibodies have been previously tested to work in ELISA, immunohistochemistry, and Western blots to confirm their specificity [6, 16, 17]. The three custom antibodies were conjugated to FITC using a conjugation kit (EasyLink, Abcam, ab102884) and stored in the dark at 4 °C. The commercially available antibody (ab91651) was conjugated to FITC or APC using a conjugation kit (EasyLink, Abcam, ab102884) and stored in the dark at 4 °C. We used FITC-conjugated CB1, A72, A74, and APC-conjugated ab91651 for flow cytometry, FITC-conjugated A72 for confocal microscopy, and unconjugated ab91651 was used for Western blotting and immunohistochemistry.

### Flow cytometry

Cells were rinsed with Dulbecco’s phosphate-buffered saline (DPBS) and treated with Accutase (Stem Cell Technology, Vancouver, Canada) at 37 °C for 5–10 min to allow for detachment and then rinsed with their respective complete medium. Cells were pelleted and resuspended at 1 × 10^6^ cells/mL in Cell Staining Buffer (BioLegend, San Diego, CA) and 200 μL of cells were placed in individual microcentrifuge tubes and stained with 1 μg of FITC-conjugated CB1, A72, A74, or APC-conjugated ab91651 for 30 min on ice in the dark. Negative controls used were unstained cells, cells stained with isotype mouse and rabbit antibodies, and NFkB to confirm the integrity of the cell membrane. Cells were then washed with Cell Staining Buffer and resuspended in 500 μL of FACS buffer. FACS buffer was made with phosphate-buffered saline (PBS), 2% calf serum (Thermo Fisher, Waltham, MA), 1 mM EDTA (Thermo Fisher, Waltham, MA, CAS 6381-92-6), and 0.1% sodium azide (Sigma Aldrich, St. Louis, MO, CAS 26628-22-8). We collected 1 × 10^4^ events per sample in a flow cytometer (Attune, Life Technologies, Carlsbad, CA) and data was analyzed using the FlowJo software (FlowJo, Ashland, OR).

### Confocal microscopy

Cells were grown on glass coverslips for 48 h. Coverslips containing cells were washed in DPBS and then incubated with either isotype control, NFkB, or A72-FITC for 30 min at 4 °C on a rocking platform. Coverslips were then washed 3 times with DPBS for 10 min on a rocking platform. We then dipped the coverslips in 1× CellMask™ Deep Red Plasma membrane stain (Thermo Fisher Scientific, Waltham, MA) for 10 min at 37 °C. The cells were rinsed in DPBS and imaged immediately using an Olympus FluoView FV1000 confocal laser scanning microscope (Olympus, Tokyo, Japan). Images were obtained using the Laser Sharp Computer Software (Bio Rad Laboratories, Hercules, CA) and later processed in Photoshop (Adobe Systems, San Jose, CA).

### Cell surface protein isolation and Western blotting

To isolate the cell surface proteins, we used the Pierce™ Cell Surface Protein Isolation Kit (Thermo Fisher Scientific, Waltham, MA, Cat # 89881). Briefly, the HT-29 cell line was grown to 90% confluency in T75 flasks and their media was removed. The cells were washed with ice-cold PBS (provided in the kit) and then the PBS was removed within 5 s. To label the cells with biotin, cells were incubated with a biotin solution made by dissolving the contents of one vial of Sulfo-NHS-SS_Biotin (provided in the kit) in 48 mL of ice-cold PBS. 10 mL of the biotin solution were added to each flask of cells and the flasks were incubated on a rocking platform with gentle agitation for 30 min at 4 °C. After incubation, 500 μL of quenching solution (provided in the kit) were added to each flask to quench the reaction. Then, cells were gently scraped and transferred to a conical tube and pelleted at 500×*g* for 3 min, after which the supernatant was discarded. Cells were then washed with 5 mL of Tris-buffered saline (TBS, provided in the kit) by pipetting up and down twice with a serological pipette and pelleted at 500×*g* for 3 min. A cocktail of protease inhibitors (Halt™ Protease & Phosphatase Inhibitor Cocktail, Thermo Fisher, Waltham, MA, product # 78440) was added to 500 μL of lysis buffer (provided in the kit) and added to the cell pellet. The cells in lysis buffer were transferred to a microcentrifuge tube and resuspended in the fluid by pipetting up and down. The cells were then disrupted using a cell disruptor (Sonicator 3000, Misonix, Inc., Farmingdale, NY) at low power (1.5) on ice using five 1-s bursts. Cells were incubated for 30 min on ice, vortexed every 5 min for 5 s, and sonicated for 1 s at low power (1.5) every 10 min. The cell lysate was centrifuged at 10,000×*g* for 2 min at 4 °C and the clarified supernatant transferred to a new microcentrifuge tube. To isolate the biotin-labeled proteins, the clarified supernatant was added to a column that contained 500 μL of NeutrAvidin Agarose that had been previously washed with 500 μL of wash buffer and centrifuged for 1 min at 1000×*g*. The column was capped and incubated for 60 min at room temperature with end-over-end mixing using a rotator. Then, the column was centrifuged for 1 min at 1000×*g* and the flow-through was discarded. The column was washed 3 times with 500 μL of wash buffer containing a cocktail of protein inhibitors to remove any other cytoplasmic proteins. To elute the membrane proteins, a 50 mM dithiothreitol (DTT) solution was made by adding 23.7 μL of 1 M DTT (provided in the kit) to 450 μL SDS-PAGE sample buffer. 450 μL of DTT solution were added to the column and incubated for 60 min at room temperature with an end-over-end mixing on a rotator. The column was then centrifuged for 2 min at 100×*g* and the flow-through collected and stored at − 20 °C. For Western blot analysis, samples were thawed on ice after which they were boiled for 5 min and then run on a 12% acrylamide SDS gel at 90 V for 2–3 h. The proteins on the gel were transferred onto a nitrocellulose membrane at 90 V for 50 min in a cold room. The nitrocellulose membrane was blocked with 5% non-fat milk in DPBS for 1 h at 4 °C in a rotating platform and then incubated in anti-TK1 commercial antibody (ab91651) in a 1:1000 dilution in milk overnight at 4 °C in a rotating platform. The nitrocellulose membrane was washed 3 times in DPBS for 3 min each in a rotating platform and then incubated with an IRDye 800 donkey anti-rabbit secondary antibody for 1 h at 4 °C in a rotating platform (LI-COR, Lincoln, NE). Finally, the membrane was washed 3 times with DPBS and imaged in an Odyssey CLx Imaging System (LI-COR, Lincoln, NE).

### Tissue dissociation and analysis

Healthy and malignant colon tissues were obtained from Utah Valley Regional Medical Center in Provo, UT under informed consent and following a protocol established by Utah Valley Regional Medical Center. Tissues were minced into 3–4 mm pieces with a sterile scalpel. Minced tissue was washed with 1× Hank’s Balanced Salt Solution (HBSS) (Thermo Fisher, Waltham, MA) containing 5% FBS. Collagenase type II or type IV (both from Thermo Fisher, Waltham, MA, product # 17101015 and # 17104019, respectively) was added to the minced tissue and incubated at 37 °C for 4–8 h to allow for cell dissociation. To obtain a cell suspension and separate dispersed cells from larger tissue pieces, cells were filtered through a 100 μm nylon mesh cell strainer (BD Biosciences, San Jose, CA). To prepare cells for flow cytometry analysis, cells were washed twice with HBSS and then resuspended in Cell Staining Buffer. Cells were treated with Fc block (Human TruStain FcX™, BioLegend, San Diego, CA), anti-human CD45 antibody (clone 2D1, eBioscience, San Diego, CA) and PI to gate out resident lymphocytes and dead cells. We collected 2 × 10^4^ events per sample in a flow cytometer (Attune, Life Technologies, Carlsbad, CA) and data was analyzed using the FlowJo software (FlowJo, Ashland, OR).

### Analysis of RNA expression data

First, we evaluated differences in expression levels of the TK1 gene in 2645 tumor samples and 264 normal samples from The Cancer Genome Atlas [18]. We used RNA-sequencing data that had been summarized at the gene level to transcripts-per-million units and processed using the *featureCounts* algorithm [19, 20]. We also log-transformed the data values. These data included expression values from tumor-adjacent and blood samples; these samples were often, but not necessarily, matched to the tumor samples. Second, we evaluated breast-cancer samples from The Cancer Genome Atlas for which hormone-receptor status had been determined via immunohistochemistry; we limited this analysis to tumors that either were (1) positive for HER2 expression or (2) negative for HER2, ER, and PR expression. In calculating differences in expression between these groups, we used a two-sided, Mann–Whitney U Test. We also evaluated the level of correlation between TK1 and six stemness and EMT genes (CD44, SNAI1, SNAI2, TWIST1, ZEB1, TGFB1) using Spearman’s method. We wrote scripts in the Python programming language (https://python.org, v.3.6.1) to parse and prepare the data. We used the R (v.3.2.2) statistical software and the ggplot2 software package (v.2.2.1) to generate graphs illustrating these expression levels [21, 22].

### Immunohistochemistry

Lung, breast, and colorectal tissue microarrays were purchased from US Biomax (Rockville, MD, catalog # LC2084, #BR721, and #CO1002b). Each slide contains at least 40 tissue cores with tissues varying in grade and stage and, and normal healthy tissues. The slides were incubated in Histo-Clear (National Diagnostic, Atlanta, GA) for 10 min for three changes to remove paraffin. The slides were then incubated in 100% EtOH for two changes for 3 min each, then in 90% EtOH for two changes for 1 min each, then in 70% EtOH for one change for 1 min. The slides were then washed in ddH_2_O for 5 min. To perform antigen retrieval, the slides were incubated in DIVA Decloaker (Biocare Medical, Pacheco, CA) at 80–95 °F for 30 min and let them cool for 10 min. We then washed 2× in ddH_2_0 for 5 min. To perform peroxidase quenching, the slides were washed in Tris-buffered saline (TBS) containing 3% hydrogen peroxide for 20 min and then washed 2× with TBS-T for 3 min. The slides were blocked for 15 min using a Background Sniper Block (Biocare Medical) to reduce background and then washed with TBS twice for 3 min. We incubated the slides with a 1:200 dilution of anti-TK1 (Abcam, ab91651) anti-GAPDH (positive control, mouse monoclonal, Cell Signaling Technologies), and universal negative control serum (negative control, Biocare Medical) and put them in a humidity chamber to prevent drying. After primary antibody staining, the slides were washed 3× for 5 min with TBS and incubated with MACH 4 Universal Horseradish Peroxidase (HRP) Polymer (universal for rabbit/mouse secondary, Biocare Medical) for 30 min in a humidity chamber and then washed again 2× for 3 min with TBS. We developed the slides with ImmPACT DAB Peroxidase (HRP) substrate (Vector Laboratories, Burlingame, CA) and then washed them 2× for 3 min with TBS. We then stained the slides with Hematoxylin (Biocare Medical) and rinsed them for 5 min in running water. We mounted the slides using cover slips and Cytoseal (Thermo Scientific) and imaged them using a light microscope. Images were analyzed using ImageJ open source software, using the “IHC (more brown)” plug-into obtain quantification using a gray scale [23]. The lower the gray value, the darker the tissue is stained.

### Statistical analysis

For our flow cytometry data, we used a multiple-comparisons one-way ANOVA test using the Sidak’s correction for multiple comparisons to compare the expression of TK1 on the surface of lung, breast, and colon cell lines vs. isotype control. All samples were compared against isotype mouse IgG except for Commercial, which was compared against isotype rabbit IgG. We set a significant P-value at ≤ 0.05. Error bars represent the standard error of the mean.

For our IHC analysis, we used Tukey’s multiple comparison test to compare the means of each group to one another. One side P-value was set to ≤ 0.05. Error bars represent the standard deviation of the samples. We used Prism 7 (GraphPad, La Jolla, CA) to perform our statistical analysis and produce graphs for most analysis. We the R (v.3.2.2) statistical software and the ggplot2 software package (v.2.2.1) to generate graphs for TK1 gene expression bioinformatics analysis.

## Results

### TK1 expression is elevated on the surface of NCI-H460, A549, MCF7, MDA-MB-231, SW620, and HT-29 cells

**Fig. 1:**
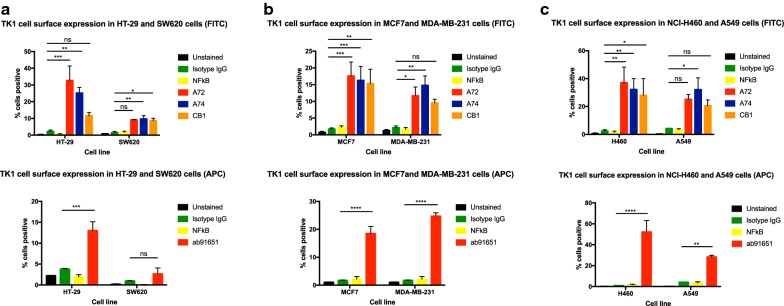
Membrane TK1 expression in of colon, breast, and lung cancer cell lines. Flow cytometry analysis of cell lines treated with anti-TK1 antibodies. **a** Quantification of TK1 expression on the cell membrane of HT-29 and SW620 cell lines stained with FITC or APC-conjugated anti-TK1 antibodies. **b** Quantification of TK1 expression on the cell membrane of MCF7 and MDA-MB-231 cell lines. The top bar graph shows MCF7 and MDA-MB-231 cell lines stained with FITC or APC-conjugated anti-TK1 antibodies. **c** Quantification of TK1 expression on the cell membrane of NCI-H460 and A549 cell lines stained with FITC or APC-conjugated anti-TK1 antibodies. Statistical analysis was performed by comparing the mouse isotype control fluorescent levels to those of A72, A74, CB1, or ab91651. *P ≤ 0.05; **P ≤ 0.005; ***P ≤ 0.001; ns = P > 0.05

Using flow cytometry, we observed an overall increase in fluorescence intensity in NCI-H460, A549, MCF7, MDA-MB-231, SW620, and HT-29 cells (Fig. 1, Additional file 1). The data represents eight independent staining procedures. Cells stained with A72 showed a significant increase in fluorescence in all cell lines but SW620 and A529 (*P ≤ 0.05; **P ≤ 0.005; ***P ≤ 0.001). Cells stained with CB1 showed significant increase in fluorescence in all cells but MDA-MB-231 and A549 (Fig. 1a–c) (*P ≤ 0.05; **P ≤ 0.005; ***P ≤ 0.001; ns = P > 0.05). Cells stained with ab91651 all showed to have an increase in fluorescence except SW620 cells (*P ≤ 0.05; **P ≤ 0.005; ***P ≤ 0.001; ns = P > 0.05). Overall, cells bound to CB1 had the lowest increase in fluorescent intensity. We used an anti-NFkB antibody as a non-specific control as well as an intracellular control. It allowed us to test whether the cells were intact and the fluorescent intensity change was due to punctured/dead cells or intact cells.

Data revealed that the lung cell lines NCI-H460 and A549 had the highest TK1 surface expression out of all the other cell lines, followed by breast cell lines MDA-MB-231 and MCF7, and colorectal cell line HT-29 (Fig. 1b, c). The SW620 cell line showed very little expression of TK1 on its surface, only and average of 10% of fluorescence total increase in cells bound to A72, A74, and CB1, and only 2.7% in cells bound to commercial antibody ab91651 (Fig. 1a). These results suggest that five of the six cell lines tested expressed TK1 on their cell surface.

### TK1 is strongly associated with the membrane of NCI-H460, MDA-MB-231, and HT-29 cells

**Fig. 2:**
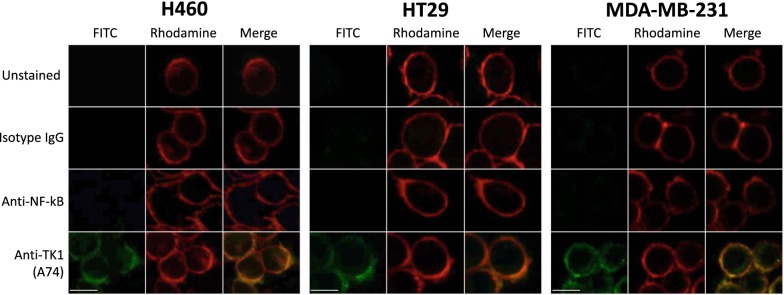
TK1 co-localizes with the plasma membrane of lung, breast, and colon cell lines. NCI-H460, HT-29, and MD-MBA-231 cells were stained with FITC-conjugated antibodies and a cell membrane specific dye (Rhodamine). Unstained cells, cells stained with isotype antibody, and cells stained with anti-NFkB antibody (FITC) were used as controls to determine the viability of cells and the non-internalization of the antibodies to prevent false positives. FITC images show staining on cells treated with anti-TK1 antibody A74. The FITC images were merged with the Rhodamine images, which show the plasma membrane. The images show a clear co-localization of TK1 with the plasma membrane of these cells

To visualize TK1’s localization and to ensure antibody binding was to the membrane only, we performed confocal microscopy in HT-29, MDA-MB-231, and NCI-H460 cells since these cell lines expressed high levels of TK1 on their membrane in flow cytometry. We stained the cells with isotype control, anti-NFkB, and anti-TK1 (A74) antibodies conjugated to FITC. We used intact cells to ensure FITC signals were not coming from intracellular TK1. We obtained single channel images, rhodamine for membrane, FITC for isotype IgG, A74 and NFKB antibodies, and overlaid them to observe associations between the signals. We observe minimal FITC signals for cells stained with isotype IgG and NFkB. However, we observe a much stronger signal from cells treated with anti-TK1 antibody (A74) (Fig. 2). These images show a clear colocalization of TK1 antibody signal and membrane dye, confirming the presence of TK1 on the surface of HT-29, MDA-MB-231, and NCI-H460 cells.

**Fig. 3:**
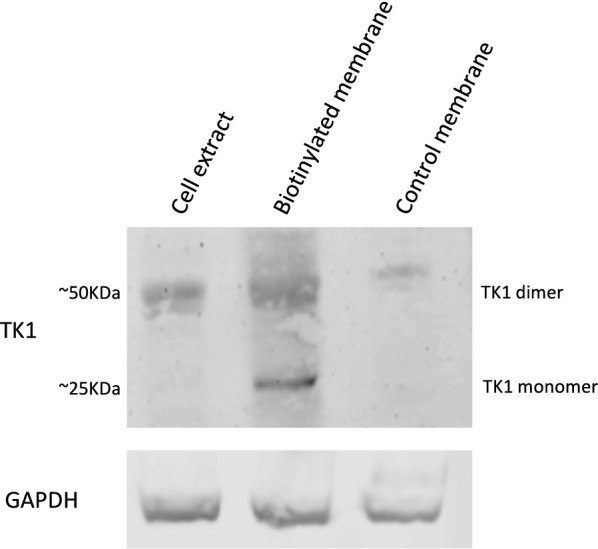
Western blot of membrane proteins isolated from HT-29 cells probed with anti-TK1 antibody ab91651. Membrane proteins from HT-29 were biotinylated and isolated for Western blot analysis. Controls included membrane protein isolation from non-biotinylated cells and cell extracts. The results show TK1 is found in the membrane of HT-29 cells in monomeric (~ 25 kDa) and dimeric forms (~ 50 kDa)

Additionally, plasma membrane proteins were isolated from the HT-29 cell line. The membrane proteins and cell extract were run on a 12% acrylamide SDS gel and then transferred to a nitro cellulose membrane and probed with anti-TK1 antibody (ab91651). We observe that TK1 is found in the membrane protein fraction of these cells further confirming the localization of TK1 on the membrane (Fig. 3). Moreover, the plasma membrane and cytosolic protein fractions show oligomeric forms of TK1 (dimer and tetramer).

### Membrane TK1 expression is significantly lower in normal colon than malignant colon clinical samples

**Fig. 4:**
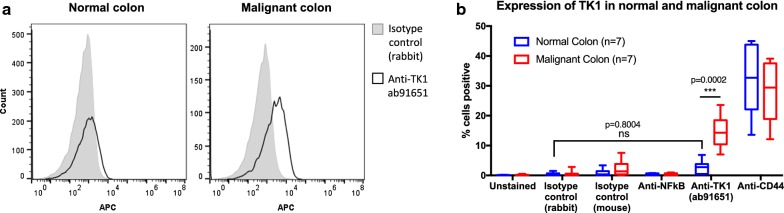
Flow cytometry evaluation of TK1 expression on the surface of colorectal patient tissue. Healthy normal and malignant colon tissue was dissociated into a single cell suspension solution and stained with anti-TK1 antibody 91651, isotype control, anti-CD44 (positive control), and anti-NFkB (negative control). Unstained cells were also used as a negative control. **a** Flow cytometry histograms showing normal and malignant colon tissue stained with isotype control (gray area) and anti-TK1 antibody ab91651 (black line). There is a definitive shift in fluorescence in the malignant cells treated with ab91651, whereas there is no shift in fluorescence in the normal colon cells treated with the same antibody. **b** Quantification and analysis of flow cytometry data on the expression of TK1 on the surface of normal and malignant colon. Malignant colon tissues show a significantly higher expression of TK1 on their membrane compared to normal colon tissues (P = 0.002). Normal colon tissues show comparable results to those of the isotype control (P = 0.8004), suggesting TK1 expression on the surface of normal colon tissues is negligible

To maintain a healthy gastrointestinal health, the lining of the gastrointestinal track needs to undergo constant cell proliferation [24]. Since TK1’s levels are proliferation-dependent, we wanted to test whether healthy normal colon cells expressed TK1 on their membrane to ascertain the clinical relevance of TK1 as biomarker target in colorectal cancer patients. We stained dissociated healthy and malignant colon tissue with anti-human CD45 antibody and PI to gate out resident lymphocytes and dead cells, and with anti-TK1 ab91651 and CD44 (positive control, adhesion protein) antibodies to test for surface expression of these two proteins. Flow cytometry revealed that the healthy normal colon tissue we tested (n = 7) showed negligible expression of TK1 on the membrane when compared to isotype control (rabbit) (P = 0.8004). Malignant colon tissue (n = 7), however, showed significantly higher expression of TK1 on the membrane compared to normal colon tissue (P = 0.0002) (Fig. 4). CD44 expression levels were not significantly different between healthy normal and malignant colon tissues (P = 0.6634). These results are crucial in establishing clinical relevance of TK1’s localization on the membrane as a unique event in cancer and not a proliferation-dependent event.

### TK1 levels are significantly higher in malignant vs. normal healthy tissue

**Fig. 5:**
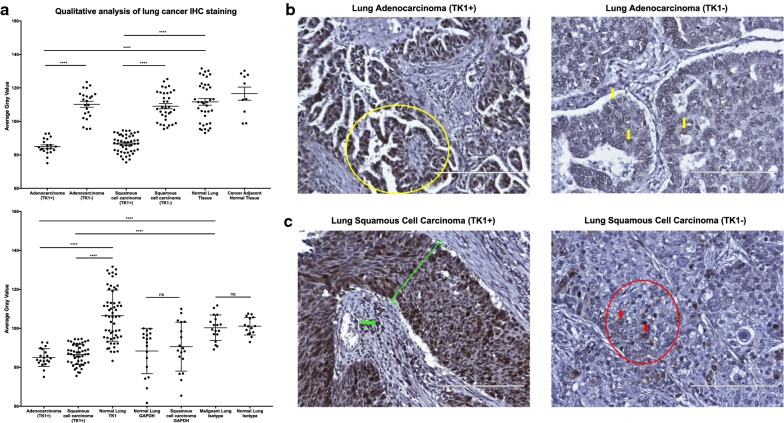
Immunohistochemistry analysis of TK1 expression in lung cancer tissue. Lung cancer tissue arrays were stained with anti-TK1 antibody ab91651, GAPDH, or isotype antibody. Tissues were imaged in a light microscope at 20×. Analysis was conducted using a gray scale. The lower the gray value, the darker the staining. **a** Quantitative analysis of lung cancer IHC staining. The top graph shows that there is a statistically significant expression of TK1 in ~ 53% of the lung adenocarcinoma tissues and in ~ 58% of the lung squamous cell carcinoma tissues. The bottom graph shows the TK1 expression next to GAPDH and isotype controls. Background levels show no statistical difference between malignant and normal healthy tissues. Malignant and normal healthy tissue showed non-statistical difference in GAPDH expression, whereas TK1 expression did show a statically significant difference between TK1+ and TK1− tissues. **b** Images showing lung adenocarcinoma tissue positive and weakly positive for TK1, which we classified as TK1− with gray value quantification. The yellow circle in adenocarcinoma TK1+ image corresponds to lung papillary adenocarcinoma formed by abnormal proliferation of glanduli form structures of papillary disposition. The adenocarcinoma TK1− image shows pulmonary adenocarcinoma tissue with acinar pattern conformed by cells of convoluted nuclei, irregular membrane, and prominent central macronucleoli. The yellow arrows correspond to a diffuse weak positive nuclear staining for TK1. **c** Images showing lung squamous cell carcinoma tissue positive and weakly positive for TK1, which we classified as TK1− with gray value quantification. In the squamous cell carcinoma TK1+ image, the green line shows strong diffuse positive nuclear staining for TK1. The green arrow shows cytoplasmic background where there is evidence of infiltration in the underlying stroma shown by positive immunostaining against TK1. In the squamous cell carcinoma TK1− image, the red circle and arrows show weakly positive nuclear focal staining in poorly differentiated lung squamous carcinoma with solid pattern. ***P ≤ 0.001; ns = P > 0.05

**Fig. 6:**
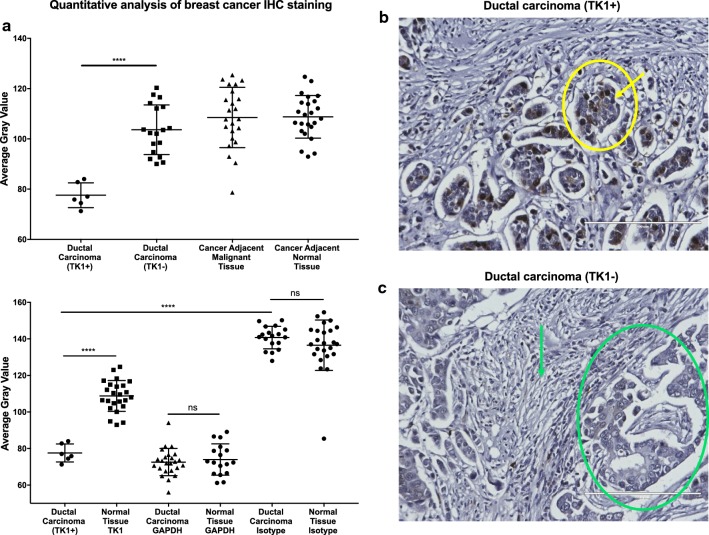
Immunohistochemistry analysis of TK1 expression in breast cancer tissue. Breast cancer tissue arrays were stained with anti-TK1 antibody ab91651, GAPDH, or isotype antibody. GAPDH was used as a positive control to account for housekeeping gene expression. The isotype antibody was used to account for background noise and non-specific binding. Tissues were imaged in a light microscope at 20×. Analysis was conducted using a gray scale. The lower the gray value, the darker the staining. **a** Quantitative analysis of breast cancer IHC staining. The top graph shows that there is a statistically significant expression of TK1 in 20% of the ductal carcinoma tissues. The bottom graph shows the TK1 expression next to GAPDH and isotype controls. Background levels show no statistical difference between malignant and normal healthy tissues. Malignant and normal healthy tissue showed non-statistical difference in GAPDH expression, whereas TK1 expression did show a statically significant difference between TK1+ and TK1− tissues. **b** Image showing breast ductal carcinoma positive for TK1. The yellow circle encloses a malignant gland structure corresponding to a moderately differentiated ductal carcinoma, and the arrow shows strong nuclear staining against TK1 in approximately 25% of the cells. **c** Image showing breast ductal carcinoma negative for TK1. The green circle shows an atypical gland structure corresponding to moderately differentiated ductal carcinoma negative for staining against TK1. The green arrow shows the tumor stroma conformed by fibrotic tissue also negative for TK1. Overall, the tissues shown in Fig. 5b, c show what we observed in the tissue’s average gray values represented in Fig. 5a, that some ductal carcinoma showed strong TK1 staining and some showed negative TK1 staining. ***P ≤ 0.001; ns = P > 0.05

**Fig. 7:**
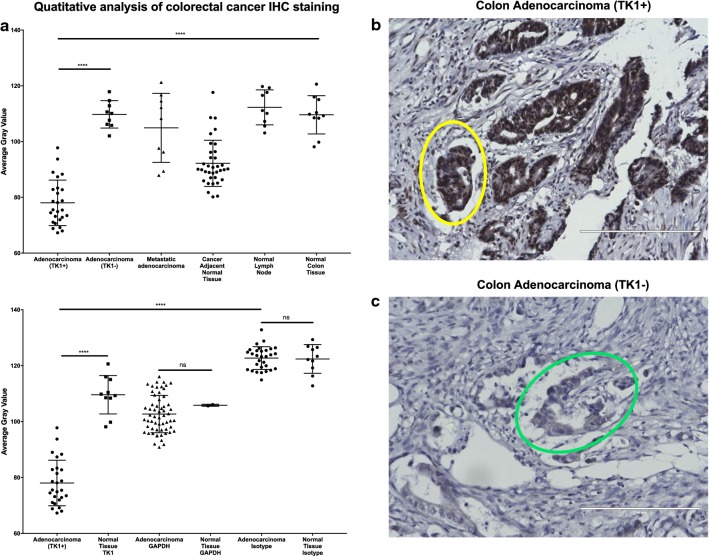
Immunohistochemistry analysis of TK1 expression in colon cancer tissue. Colon cancer tissue arrays were stained with anti-TK1 antibody ab91651, GAPDH, or isotype antibody. GAPDH was used as a positive control to account for housekeeping gene expression. The isotype antibody was used to account for background noise and non-specific binding. Tissues were imaged in a light microscope at 20×. Analysis was conducted using a gray scale. The lower the gray value, the darker the staining. **a** Quantitative analysis of colorectal cancer IHC staining. The top graph shows that there is a statistically significant expression of TK1 in ~ 74% of the colon adenocarcinoma tissues. The bottom graph shows the TK1 expression next to GAPDH and isotype controls. Background levels show no statistical difference between malignant and normal healthy tissues. Malignant and normal healthy tissue showed non-statistical difference in GAPDH expression, whereas TK1 expression did show a statically significant difference between TK1+ and TK1− tissues. **b** Image showing colorectal adenocarcinoma positive for TK1. The yellow circle encloses an atypical glandular structure positive for TK1 in over 90% of the cells. **c** Image showing colorectal adenocarcinoma negative for TK1. The circle an atypical glandular structure negative for TK1. Overall, the tissues shown in (**b**, **c**) show what we observed in the tissue’s average gray values represented in **a**, that some colorectal adenocarcinoma tissues showed strong TK1 staining and some showed negative TK1 staining. ***P ≤ 0.001; ns = P > 0.05

We stained lung, breast, and colon tissue arrays containing healthy normal, healthy normal adjacent, cancer adjacent, and malignant tissue with anti-TK1 antibodies to establish overall expression of TK1. We imaged all tissues with a light microscope at 20×. We conducted the analysis using a gray scale. The lower the gray value, the darker the staining. There was a significant increase in TK1 expression in malignant tissues compared to normal healthy tissues, where TK1 expression was negligible (Figs. 5, 6 and 7). However, there was also a portion of malignant tissues that stained negative for TK1 (Table 1). Lung tissue array shows ~ 50% of the tissues stain positive for TK1 in both adenocarcinoma and squamous cell carcinoma (Fig. 5a, Table 1). We also observe that normal cancer adjacent tissue is negative for TK1 (Fig. 5a). We can observe a clear differential expression between malignant and normal healthy tissue in both lung adenocarcinoma and squamous cell carcinoma (Fig. 5b, c). In breast tissue arrays, infiltrating ductal carcinoma positive for TK1 has a significantly lower average gray value than normal breast tissue stained for TK1 (Fig. 6a). TK1 staining localizes to the gland structures (Fig. 6a) in TK1+ tissue. Gland structures or stroma did not stain with TK1 antibody in TK1− tissue (Fig. 6b). These binary results are also observed in colorectal tissue arrays (Fig. 7). However, metastatic adenocarcinoma stained negative for TK1 (n = 9) (Fig. 7a). The darkest TK1 staining in colorectal adenocarcinoma can be found in groups of atypical glandular structures as seen in Fig. 7b. TK1− colorectal adenocarcinoma tissue shows no staining in the glandular structures (Fig. 7c).

**Table 1:** TK1 staining in normal and malignant tissue

Tissue type	Negative	Positive
Lung adenocarcinoma	20	23
Lung squamous cell carcinoma	35	94
Normal lung	40	0
Cancer adjacent normal lung	10	0
Breast ductal carcinoma	6	19
Normal breast	24	0
Colon adenocarcinoma	9	26
Normal colon	10	0

### TK1 gene expression levels are upregulated in malignant lung adenocarcinoma, lung squamous carcinoma, breast invasive carcinoma, and colorectal adenocarcinoma

**Fig. 8:**
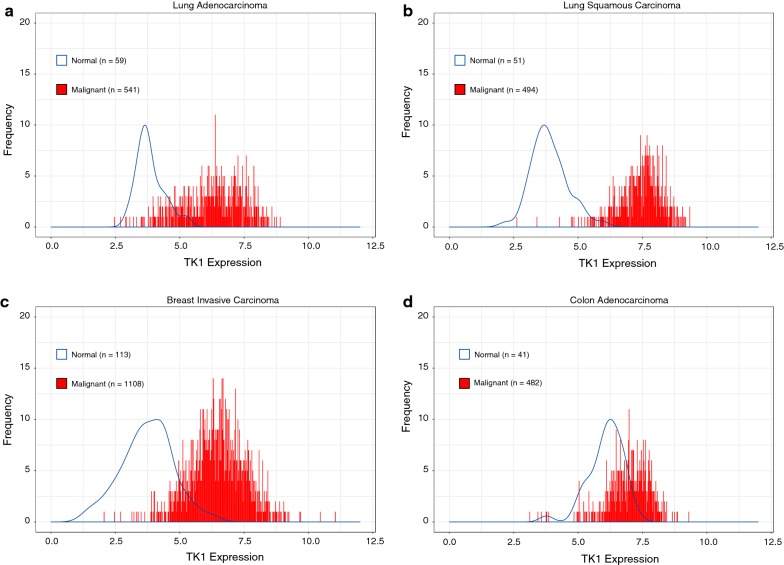
Lung, breast, and colorectal cancer patients show upregulated TK1 gene expression compared to normal patients. **a** TK1 gene expression in lung adenocarcinoma vs. normal lung patients. **b** TK1 gene expression in lung squamous cell carcinoma vs. normal lung patients. **c** TK1 gene expression in breast invasive carcinoma vs. normal breast patients. **d** TK1 gene expression in colon adenocarcinoma vs. normal colon patients

We also evaluated TK1 expression using RNA-Sequencing data from The Cancer Genome Atlas (TCGA). Our analysis reveals that TK1 levels are upregulated in both lung adenocarcinoma and lung squamous cell carcinoma, breast invasive carcinoma, and colorectal adenocarcinoma patients (Fig. 8). Lung adenocarcinoma and lung squamous carcinoma seem to be the malignancies with the most differential expression of TK1 between normal and malignant patients, followed by breast invasive carcinoma, where we could also observe clear differential expression (Fig. 8a–c). There was some overlap in TK1 expression in colorectal adenocarcinoma patients vs. normal patients, probably due to the highly proliferative nature of the colon, as TK1 levels rise during proliferation. However, TK1 levels in colorectal adenocarcinoma patients are still more upregulated than in healthy normal patients (Fig. 8d).

### Triple negative breast cancer patients show higher levels of TK1 than HER2+ cancer patients

**Fig. 9:**
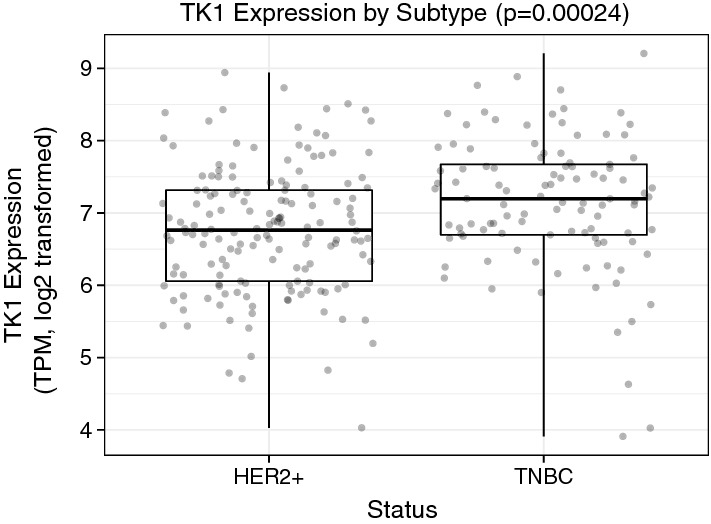
Boxplot of RNA-Sequencing data from The Cancer Genome Atlas showing TK1 gene expression in HER2+ breast tumors and triple negative breast cancer (TNBC) tumors. The boxplot shows there is a significant upregulation in TK1 expression in TNBC tumors compared to HER2+ tumors

We evaluated TK1 gene expression using RNA-sequencing data from TCGA in breast cancer patient available data for which hormone receptor status was available. Data was analyzed by tumors that were either HER2+ status or HER2- and ER-, and PR-status (triple negative breast cancer, TNBC). We found that TNBC tumor samples expressed higher levels of TK1 than HER2+ tumor samples (Fig. 9). These data seem consistent with membrane expression of TK1 in MDA-MB-231 cells (TNBC), which express higher levels of TK1 on their surface than MCF7 cells (HER2+) when stained with ab91651 antibody (Fig. 1c).

### TK1 expression levels in triple negative breast cancer show positive correlation with stem cell and EMT markers

**Fig. 10:**
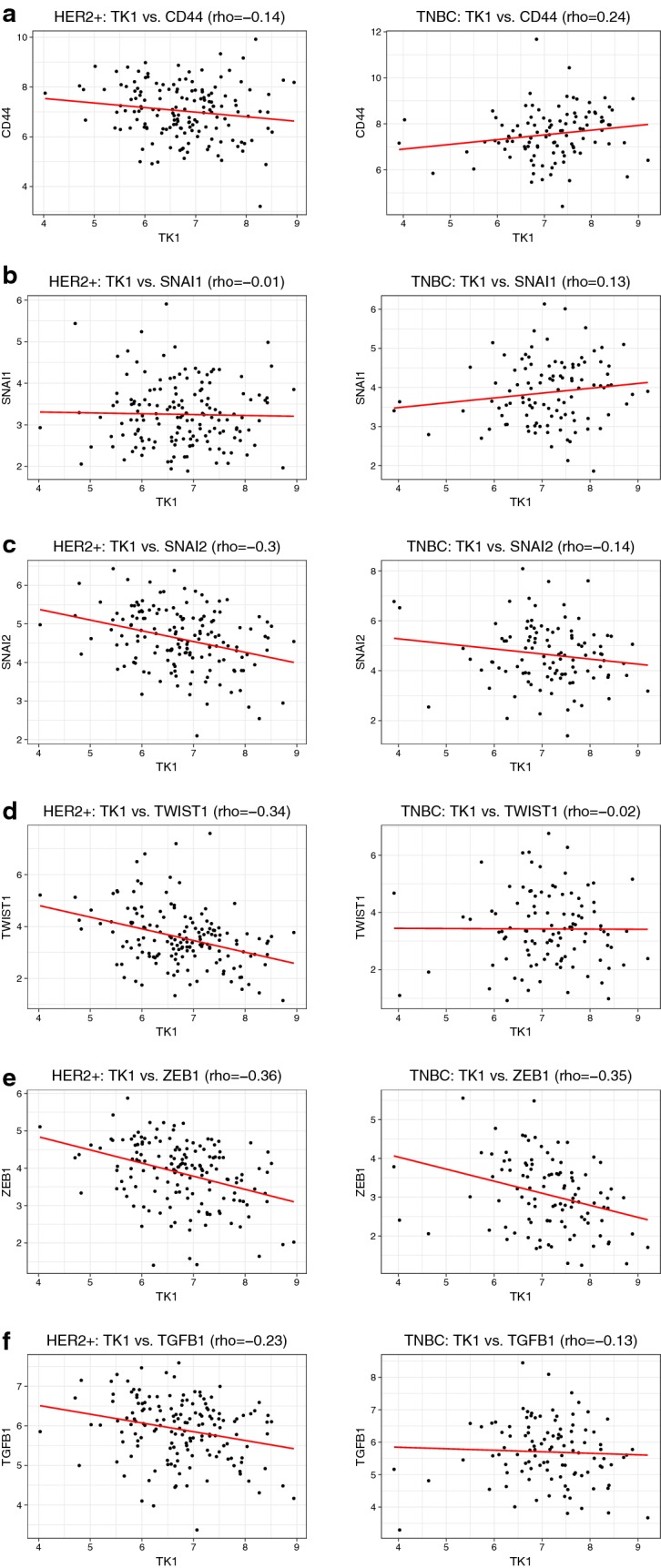
Scatterplots of RNA-sequencing data from The Cancer Genome Atlas comparing TK1 expression to six stemness and EMT markers in HER2+ breast tumors and triple negative (TNBC) tumors. **a** Cell stemness marker CD44 is positively correlated to TK1 in TNBC tumors and negatively correlated to TK1 in HER2+ breast tumors. **b** EMT marker SNAI1 is positively correlated to TK1 in TNBC tumors and negatively correlated to TK1 in HER2+ breast tumors. **c** EMT marker SNAI2 is negatively correlated to TK1 in TNBC tumors and in HER2+ breast tumors. **d** EMT marker TWIST1 is negatively correlated to TK1 in TNBC tumors and in HER2+ breast tumors. **e** EMT marker ZEB1 is negatively correlated to TK1 in TNBC tumors and in HER2+ breast tumors. **f** Stemness and EMT marker TGFB1 negatively correlated to TK1 in TNBC tumors and in HER2+ breast tumors

RNA expression analysis was performed to evaluate the level of correlation between TK1 and six stemness and EMT genes (CD44, SNAI1, SNAI2, TWIST1, ZEB1, TGFB1). We found that in TNBC TK1 expression levels positively correlated with CD44 (rho = 0.24) and SNAI1 (0.13), and negatively correlated with SNAI2 (rho = − 0.14), TGFB1 (rho = − 0.13), TWIST1 (rho = − 0.02), and ZEB1 (rho = − 0.35). On the other hand, we found that in HER2+ tumors, TK1 expression levels negatively correlated with all the markers (Fig. 10a–f). These results are interesting, as it reveals that TK1 levels may correlate with stemness and invasion potential in cancer cells.

## Discussion

The salvage pathway enzyme TK1 plays a crucial role in pyrimidine deoxynucleotide synthesis during the cell cycle. Because of this critical association to proliferation and the cell cycle, TK1 has been established as a proliferation biomarker in many cancers, including lung, breast, and colorectal. Serum TK1 (sTK1) has been used in many applications for the early detection and diagnosis of cancer, as it is found upregulated in cancer patients. In this study, we present data supporting the expression and localization of TK1 on the cellular membrane of lung, breast, and colon cancer, suggesting TK1 as a surface marker for these malignancies and a different function for TK1 never reported before. The expression of TK1 on the surface of these solid malignancies seems to mirror that of other surface/stem cell markers such as NCAM in lung cancer, CD133 in lung and colon cancer, and CD298 in breast cancer [25–28]. Our results also correlated with some of our previous findings, which show that TK1 localizes on the surface of hematological malignancies such as Burkitt’s lymphoma, acute lymphoblastic leukemia, promyelocytic leukemia, and T-cell lymphoma cells [29]. Interestingly, membrane TK1 seems to be found in monomeric and dimer form similar to membrane TK1 found in hematological malignancies, which suggest kinase enzymatic activity [29]. The actual function of membrane-expressed TK1 is still unknown, however.

Moreover, we show that in colon patient tissue, a highly proliferative tissue, TK1 is expressed on the membrane of only malignant cells and not healthy normal cells. This may mean that TK1’s localization to the cell membrane is an event unique to malignancy. We have seen similar results in the expression of TK1 in hematological malignancies vs. normal proliferating lymphocytes, where TK1 only localized to the membrane of the cancer cells [29].

We also report clinical data from The Cancer Genome Atlas (TCGA), where we explore TK1 gene expression levels, showing that TK1 levels are upregulated in lung, breast, and colorectal cancer patients compared to their healthy normal patients. The bioinformatics analysis reveals that the TK1 expression in some normal healthy tissues overlap with that of some malignant tissues. This is an important observation for clinical relevance, as the gene expression results correlate with our IHC results also shown in this study. IHC reveals that normal healthy tissue is negative for TK1, but some malignant tissue stains positive for TK1 and some malignant stains weakly positive or negative for TK1 in all lung, breast, and color tissue arrays. These results suggest that not all malignancies will have TK1 as a biomarker.

Clinically speaking, a good surface biomarker will be one that overexpresses on the membrane, shows stable expression levels in tumors, and is low or absent in normal cells/tissues [30]. For example, the Erb-B2 Receptor Tyrosine Kinase 2 (HER2) is overexpressed in subsets of breast, ovarian, gastric, colorectal, pancreatic and endometrial cancers [31]. In breast and colon cancer, HER2+ tumors are treated by targeting HER2 on the membrane [32, 33]. Current treatments include Herceptin (trastuzumab), Perjeta (pertuzumab), Tykerb (lapatinib), and Kadcyla (T-DM1 or ado-trastuzumab emtansine). Comparing HER2 and TK1, both proteins are heterogeneously expressed in cancer tissues [34, 35]. Moreover, surface expression levels of HER2+ cancer cell lines are comparable to surface expression of TK1. In fact, A549 and NCI-H460 cells lines show higher expression of TK1 on the surface to breast and colon cancer cell lines reported in the literature [36–38]. We also report that TK1 gene expression levels are significantly higher in TNBC vs. HER2+ breast tumors, suggesting TK1 as an alternative biomarker and potential target for TNBC. We also show that in TNBC tumors, TK1 levels positively correlate with two stemness and EMT markers, whereas in HER2+ tumors, TK1 levels show the opposite correlation. This suggest that increased levels of TK1 may aid or have a function in invasion and migration potential in cancer cells.

Further research is needed to help elucidate the mechanism by which TK1 reaches the cell membrane and to understand the function of TK1 on the membrane.

## Conclusions

This study shows that TK1 localizes on the cell membrane of NCI-H460, A549, MCF7, MDA-MB-231, SW620, and HT-29 cells lines and on the membrane of colorectal cancer cells and not on the membrane of healthy colorectal cells from patients. This indicates that TK1’s localization on the cell surface may be an event unique to malignancy and independent of proliferation. We also show that TK1 is upregulated in a significant population of cancer patient tissues and that TK1 gene expression is also upregulated in cancer patients compared to normal healthy patients. Given these results, TK1 could potentially be used as an immunotherapeutic target, either using antibodies against TK1, a drug-antibody conjugate, or a chimeric antigen receptor (CAR) T cell targeting TK1.

## Additional file

**Additional file 1.** Flow cytometry histograms of cell lines treated with anti-TK1 antibodies. Cells treated with anti-TK1 antibodies (black line) showed a shift in fluorescence compared to isotype controls (gray area).

## Abbreviations

CAR: chimeric antigen receptor
DPBS: Dulbecco’s phosphate-buffered saline
DTT: dithiothreitol
FBS: fetal bovine serum
IHC: immunohistochemistry
STR: short tandem repeat
TCGA: The Cancer Genome Atlas
TK1: thymidine kinase 1
