# Correction to: Membrane expression of thymidine kinase 1 and potential clinical relevance in lung, breast, and colorectal malignancies

**DOI:** 10.1186/s12935-019-0749-6

**Published:** 2019-02-11

**Authors:** Evita G. Weagel, Weston Burrup, Roman Kovtun, Edwin J. Velazquez, Abigail M. Felsted, Michelle H. Townsend, Zachary E. Ence, Erica Suh, Stephen R. Piccolo, K. Scott Weber, Richard A. Robison, Kim L. O’Neill

**Affiliations:** 1 Department of Microbiology and Molecular Biology, Brigham Young University, 3142 Life Sciences Building, Provo, UT 84602 USA; 2 Department of Biology, Brigham Young University, Provo, UT USA; 3 Department of Biomedical Informatics, University of Utah, Salt Lake City, UT USA

## Correction to: Cancer Cell Int (2018) 18:135 10.1186/s12935-018-0633-9

Following publication of the original article [1], the authors would like to make an amendment to the Acknowledgements section.

- The acknowledgments currently read:

We would like to thank the Simmons Center for Cancer Research for the financial support, Dr. Juan Arroyo (BYU) for his assistance in tissue staining and confocal microscopy, Dr. Himelda Chavez (Universidad Nacional Mayor de San Marcos, Lima, Peru), for her pathology expertise and help in IHC analysis, and a team of surgeons at Utah Valley Regional Medical Center for providing normal and malignant colon tissue.

- The corrected acknowledgements would read:

We would like to thank the Simmons Center for Cancer Research for the financial support, Dr. Juan Arroyo (BYU) for his assistance in tissue staining and confocal microscopy, Dr. Himelda Chavez (Universidad Nacional Mayor de San Marcos, Lima, Peru), for her pathology expertise and help in IHC analysis, and a team of surgeons at Utah Valley Regional Medical Center for providing normal and malignant colon tissue. Results from this study are in part based upon data generated by The Cancer Genome Atlas and managed by the United States National Cancer Institute and National Human Genome Research Institute (see http://cancergenome.nih.gov).
