# Supplementary figures and images for: Membrane expression of thymidine kinase 1 and potential clinical relevance in lung, breast, and colorectal malignancies
Source: Cancer Cell Int. 2018 Sep 10;18:135. doi: 10.1186/s12935-018-0633-9 (PMC6131957; doi:10.1186/s12935-018-0633-9)

HT-29

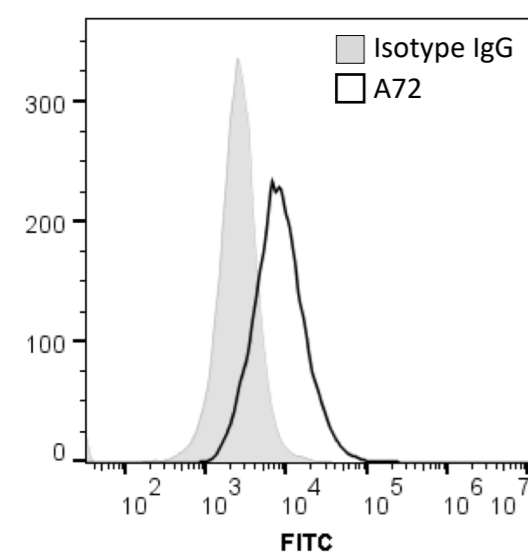

SW620

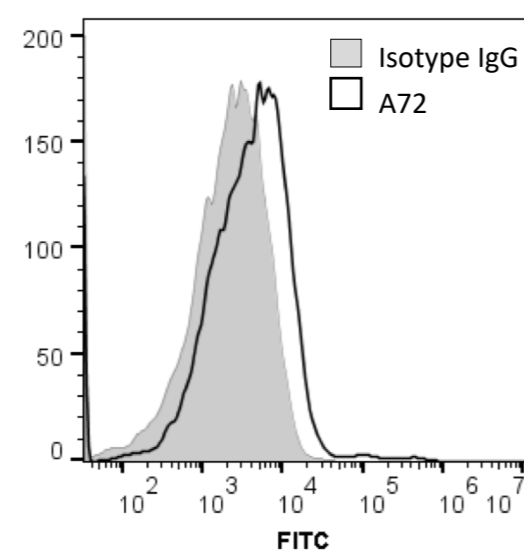

MCF7

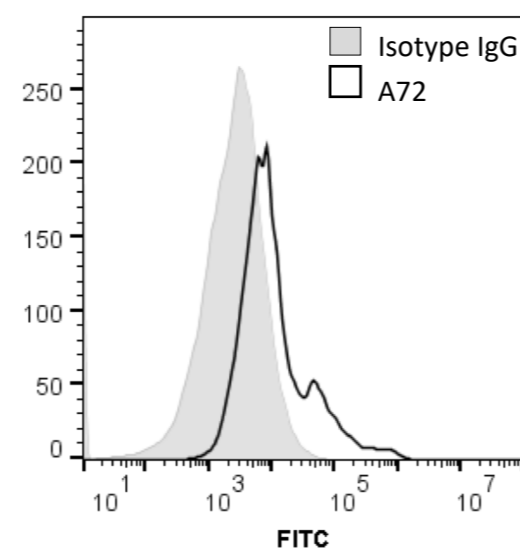

MDA-MB-231

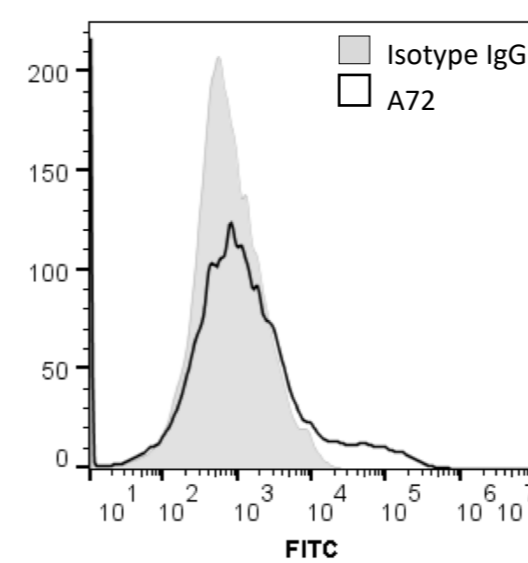

A549

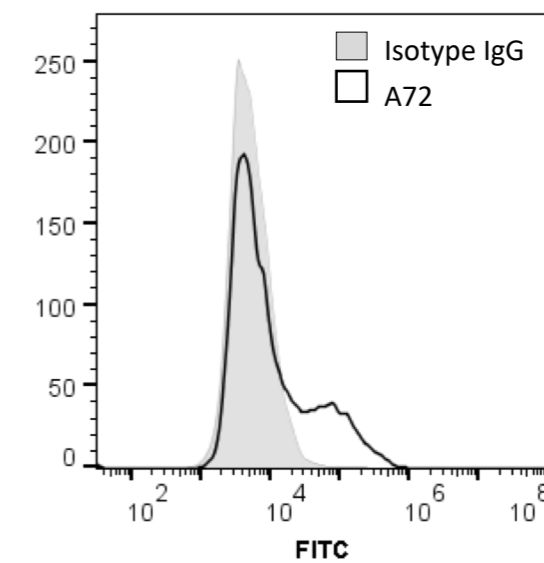

NCI-H460

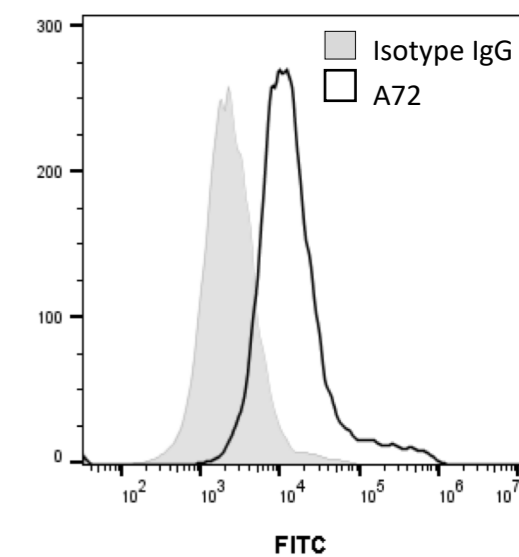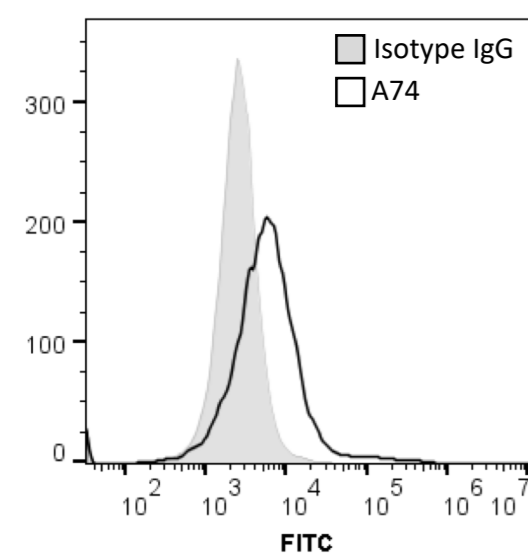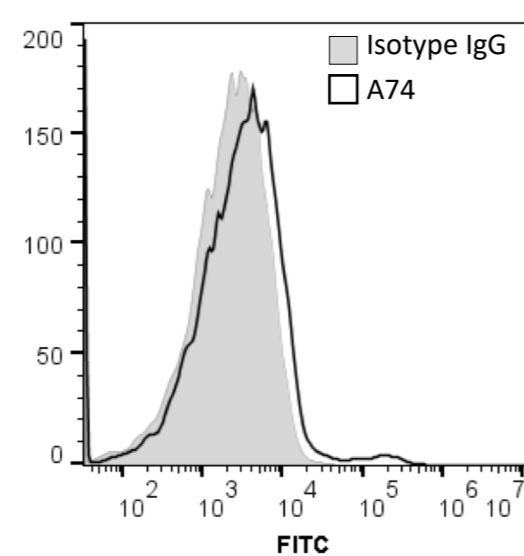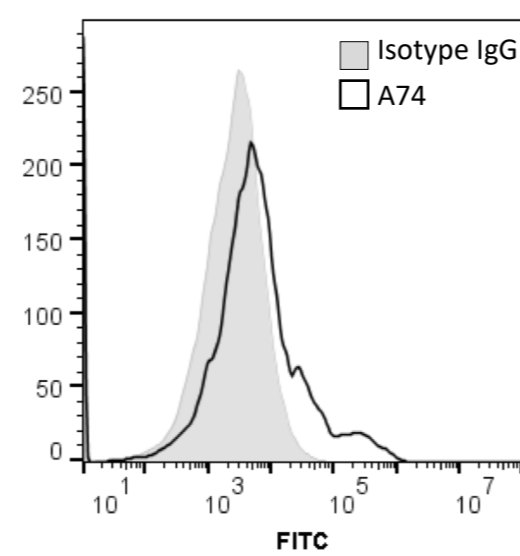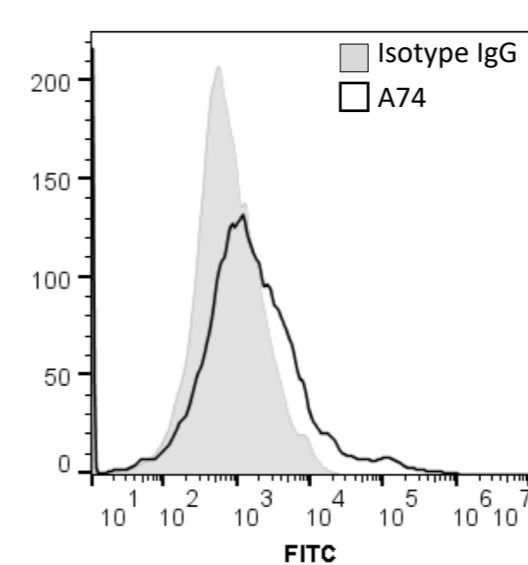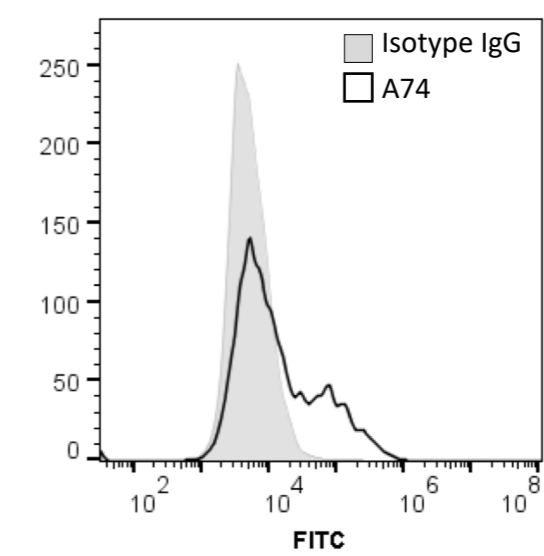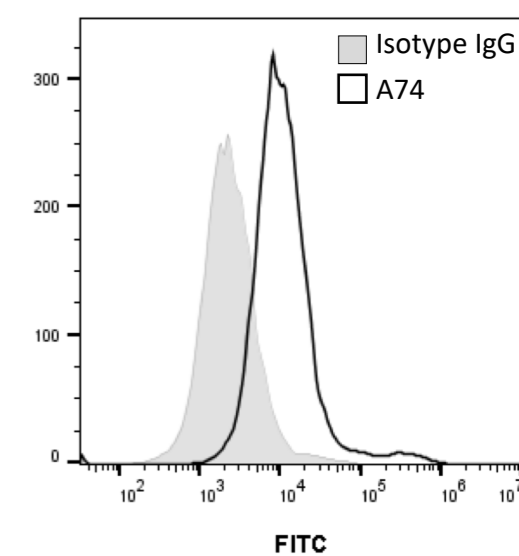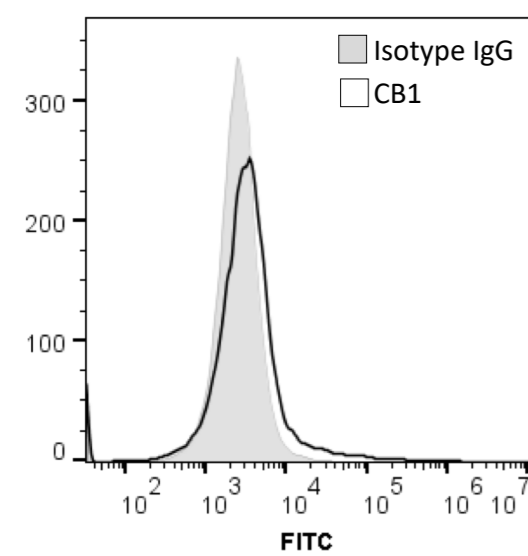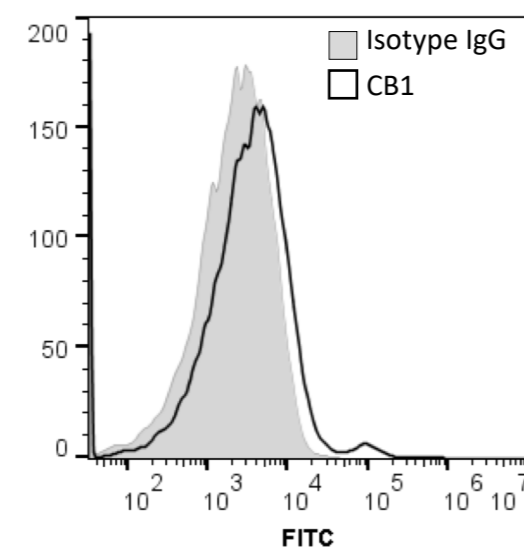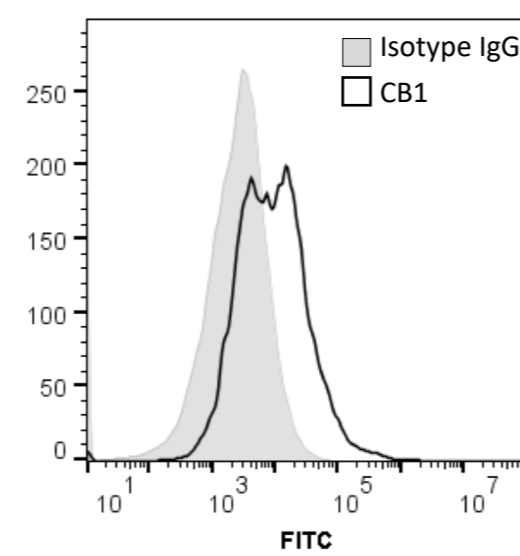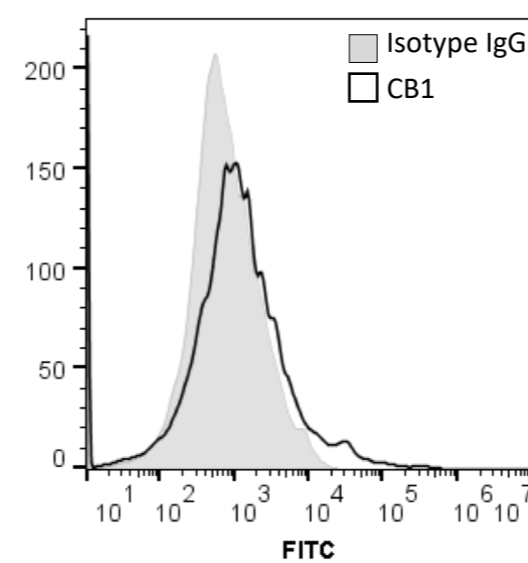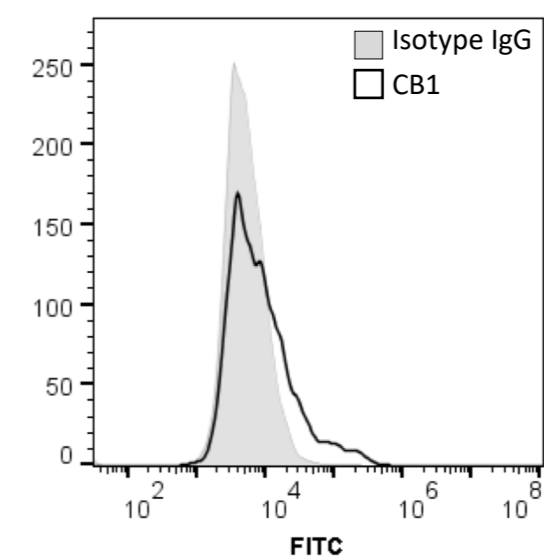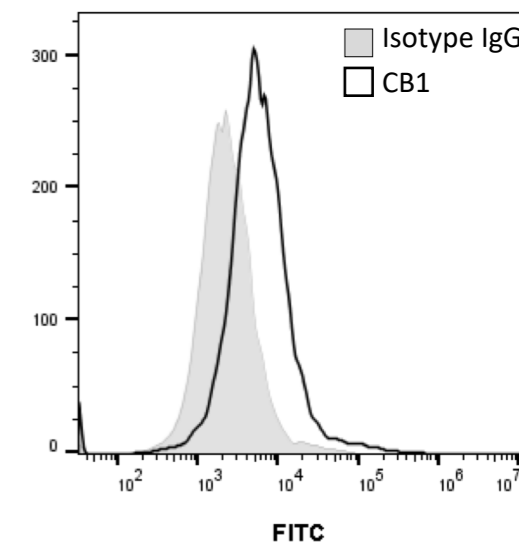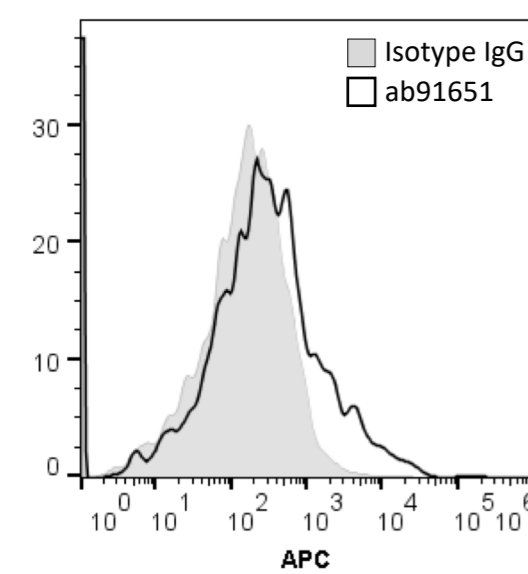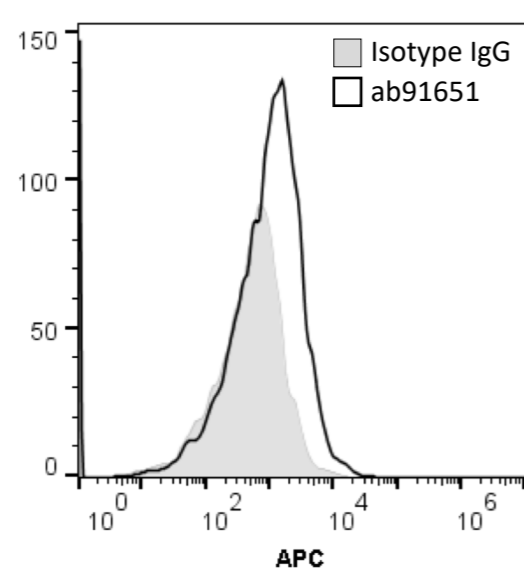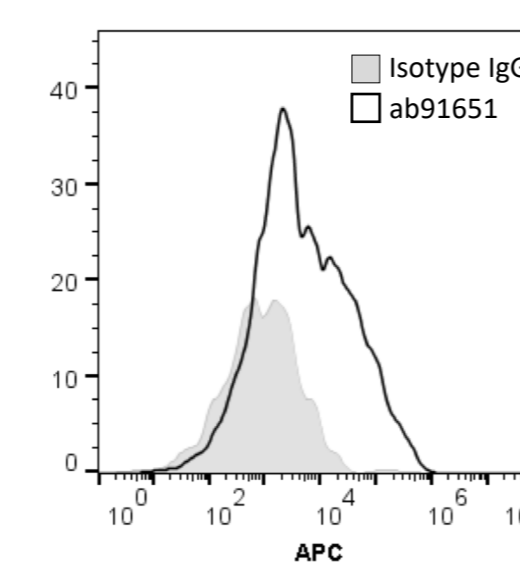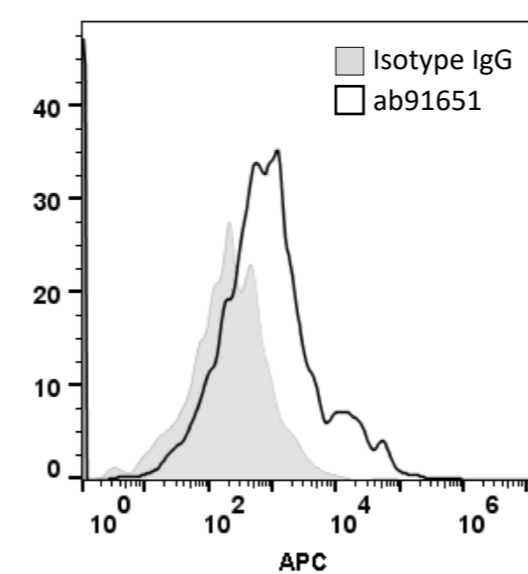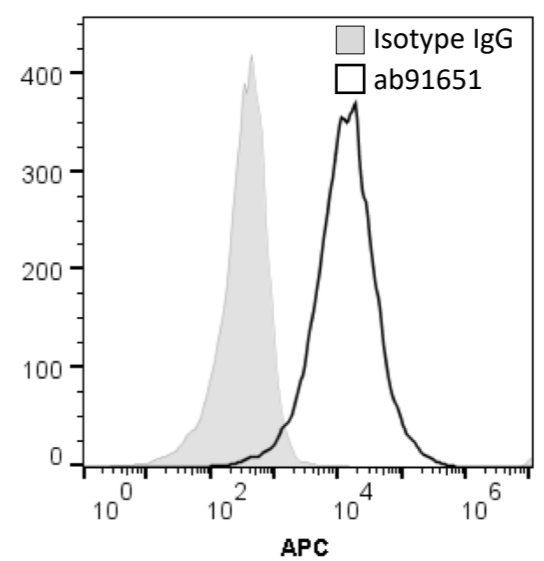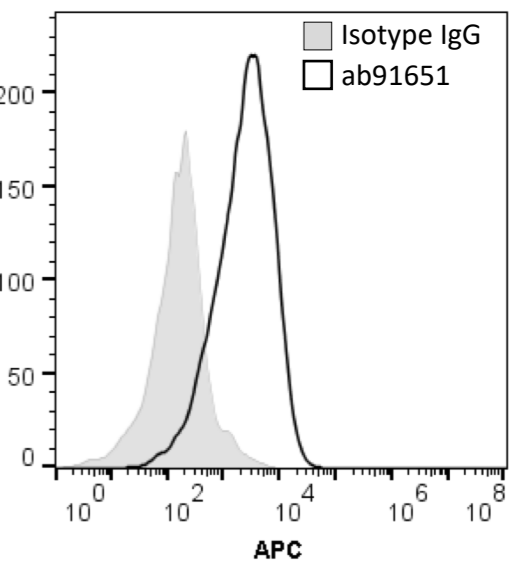

Supplement: Supplementary file 1 — Additional file 1. Flow cytometry histograms of cell lines treated with anti-TK1 antibodies. Cells treated with anti-TK1 antibodies (black line) showed a shift in fluorescence compared to isotype controls (gray area). [file 12935_2018_633_MOESM1_ESM.pdf]
